# A Therapeutic Small Molecule Enhances γ‐Oscillations and Improves Cognition/Memory in Alzheimer’s Disease Model Mice

**DOI:** 10.1002/alz.095553

**Published:** 2025-01-09

**Authors:** Xiaofei Wei, Jesus J Campagna, Barbara Jagodzinska, Dongwook Wi, Whitaker Cohn, Jessica Lee, Chunni Zhu, Christine S. Huang, László Molnár, Carolyn R. Houser, Varghese John, Istvan Mody

**Affiliations:** ^1^ The David Geffen School of Medicine at UCLA, Los Angeles, CA USA; ^2^ Drug Discovery Lab, Mary S. Easton Center for Alzheimer’s Disease Research at UCLA, Los Angeles, CA USA; ^3^ Sapientia Hungarian University of Transylvania, Târgu Mureş, Mureş Romania

## Abstract

**Background:**

Brain rhythms provide the timing for recruitment of brain activity required for linking together neuronal ensembles engaged in specific tasks. The γ‐oscillations (30‐120 Hz) orchestrate neuronal circuits underlying cognitive processes and working memory. High temporal resolution recording methods, such as magnetoencephalography, have made it clear that Alzheimer’s disease (AD) patients, starting as early as the mild cognitive impairment (MCI) stage, have diminished γ‐oscillations even before the Aβ load takes full effect. Moreover, it has been well established that almost all AD mouse models have diminished γ‐oscillations.

**Method:**

We describe a unique pharmacological approach meant to enhance cognitive performance and working memory in a state‐dependent manner by engaging and amplifying the brain’s endogenous γ‐oscillations through enhancing the function of parvalbumin positive interneurons (PV+INs). We employed anatomical, in vitro and in vivo electrophysiological, and behavioral methods to examine the effects of our lead therapeutic candidate small molecule.

**Result:**

We anatomically and pharmacologically identified GABA_A_Rs assembled of α1β2δ subunits responsible for the tonic inhibition of PV+INs. We further demonstrated that DDL‐920, a small molecule negative allosteric modulator (NAM) of these receptors, is a potent, efficacious, and selective blocker of the tonic inhibition of PV+INs and consequently enhances γ‐oscillations both in vitro and in vivo. When orally administered twice daily for two weeks, DDL‐920 restored the cognitive/memory impairments of 3‐4‐month‐old AD model mice as measured by their performance in the Barnes maze.

**Conclusion:**

Our findings indicate that the unique subunit composition of extrasynaptic GABA_A_Rs of PV+INs should be a valid target for boosting γ‐oscillations and this should be beneficial in a variety of neurological and psychiatric disorders, including AD.

